# Außerklinische Intensivpflege – Versorgungsrelevante Aspekte nach persönlichen Vor-Ort-Begutachtungen durch den Medizinischen Dienst. Ein Praxisbericht

**DOI:** 10.1055/a-2711-1073

**Published:** 2025-12-09

**Authors:** Thomas Gaertner, Andreas Mappes, Moritz Rau, Ulfert Grimm, Annette Hoffmann-Götz, Patrick Schunda

**Affiliations:** 1 Stabsstelle Sozialmedizinisches Wissens- und Qualitätsmanagement, Medizinischer Dienst Hessen, Oberursel, Germany; 2 Geschäftsbereich Ambulante Versorgung, Medizinischer Dienst Hessen, Oberursel, Germany; 3 Leitender Arzt, Medizinischer Dienst Hessen, Oberursel, Germany

**Keywords:** Medizinischer Dienst, Außerklinische Intensivpflege, Begutachtung, Beatmungsentwöhnung, Tracheostoma, Verordnung, Medical Advisory Service, out-of-hospital intensive care, assessment, weaning, tracheostomy, medical prescription

## Abstract

Die sozialgesetzlich im SGB V neu gefasste außerklinische Intensivpflege (AKI) gestattet schwerstkranken Patienten, die zum großen Teil trachealkanüliert oder beatmungspflichtig sind, ein soweit als möglich teilhabeorientiertes Leben auch in häuslicher Umgebung. Als Leistung der Krankenkassen setzt sie eine spezielle vertragsärztliche Verordnung voraus, eine qualifizierte Potenzialerhebung wird bei beatmeten bzw. trachealkanülierten Patienten zusätzlich verlangt. Eine jährlich stattfindende, obligate Begutachtung durch den Medizinischen Dienst (MD) am Leistungsort bildet zusammen mit der ärztlichen Verordnung die Grundlage der genehmigenden Leistungsentscheidung der Krankenkasse. Es wurde eine Sekundärdatenanalyse von insgesamt 1615 Vor-Ort-Begutachtungen des MD Hessen aus den ersten 17 Monaten nach Inkrafttreten der neuen Regelungen und Maßgaben zur Durchführung der AKI durchgeführt. Bei dem hohen Anteil an tracheotomierten Patienten von 72,6% der Gesamtpopulation wurden 27,7% durchgängig oder intermittierend invasiv beatmet. Bei 46,5% war eine damals noch zwingend erforderliche Potenzialerhebung noch ausstehend. In rund 8,2% aller Fälle konnte die Notwendigkeit einer AKI sozialmedizinisch nicht nachvollzogen werden. Die jährliche MD-Begutachtung vor Ort ermöglicht erstmals ein individuelleres und differenzierteres Bild der speziellen Konstellation nach ICF-Kriterien mit Blick auf die sozialmedizinische Notwendigkeit einer AKI-Versorgung. Schwerpunkte sind hier insbesondere die Sicherstellung einer adäquaten medizinischen und pflegerischen Versorgung vor Ort sowie die Ausschöpfung eines festgestellten Weaning- und Dekanülierungspotenzials.

## Abstract (en)

The new out-of-hospital intensive care (AKI) law, which is now included in social law, allows critically ill patients, most of whom are tracheostomy patients or require ventilation, to live a participatory life in a home environment as far as possible. As a service provided by statutory health insurance companies, it requires a special prescription from a statutory health insurance provider and, in the case of ventilated or tracheotomized patients, a qualified potential assessment for weaning and/or decannulation (removal of the tracheostomy tube). A mandatory assessment by the Medical Advisory Service (MAS) at the place of care is the basis for the health insurance company's decision for benefit approval. A secondary data analysis was carried out consisting of 1615 initial reports from the Hessian MAS of the first 17 months after the new regulations and requirements for AKI were implemented. Of the high proportion of tracheotomized patients (72.6%) of the total population, 27.7% received continuous or intermittent invasive ventilation. In 46.5% of cases, a potential assessment, which was mandatory at the time, was still pending. From a socio-medical perspective, in around 8.2% of all assessed cases, the AKI requirements were not met. The MAS assessment enables a more individual and differentiated picture of the special constellation according to ICF criteria with a view to the socio-medical necessity of AKI care. Furthermore, it helps to ensure adequate medical and nursing care on site and reviews any weaning potential that may have not been sufficiently exploited to this point.

## Einleitung

Gemäß dem Fünften Buch Sozialgesetzbuch – Gesetzliche Krankenversicherung (SGB V) haben Versicherte „Anspruch auf Krankenbehandlung, wenn sie notwendig ist um eine Krankheit zu erkennen, zu heilen, ihre Verschlimmerung zu verhüten oder Krankheitsbeschwerden zu lindern.“ Hierbei handelt es sich um vertragsärztlich zu verordnende ärztliche und nichtärztliche ambulante Leistungen. Diese Krankenbehandlung umfasst neben der häuslichen Krankenpflege auch die außerklinische Intensivpflege (AKI).

Mit dem am 23.10.2020 in Kraft getretenen Intensivpflege- und Rehabilitationsstärkungsgesetz (GKV-IPReG) bzw. mit Art. 2 zum 31.10.2023 wurde dem SGB V ein Anspruch auf AKI als eigenständige Rechtsvorschrift in Form des neuen § 37c hinzugefügt. Originäre gesundheitspolitische Ziele des GKV-IPReG waren eine Verbesserung der Lebens- und Versorgungsqualität der Betroffenen, zudem ein gezielteres Ausschöpfen des Weaning- bzw. Dekanülierungspotenzials bei beatmeten bzw. trachealkanülierten Versicherten, sowie eine Vermeidung von Fehlanreizen und die Beseitigung von Missständen 1 .

Die vordem gültige „Spezielle Krankenbeobachtung“ nach § 24 der Häuslichen Krankenpflege-Richtlinien (HKP-RL) ist damit seit dem 31.10.2023 ersatzlos gestrichen und nicht mehr zu verordnen. In Zuge dessen traten die neugefassten sozialrechtlichen Regelungen zur AKI als besondere Leistung der Krankenkassen in Kraft. Demzufolge können Versicherte mit einem besonders hohen Bedarf an medizinischer Behandlungspflege durch ständig anwesende, geeignete Fachpflegefachkräfte individuell überwacht werden. So wird mit der AKI auch schwerstkranken, zum großen Teil beatmungspflichtigen oder trachealkanülierten Patientinnen und Patienten ermöglicht, ein teilhabeorientiertes Leben in häuslicher Umgebung zu realisieren. Zudem können die häufig multimorbiden Menschen in Abhängigkeit vom Schweregrad der zugrundeliegenden Erkrankung oft ein nachweislich höheres Lebensalter erreichen 2 .

AKI ist indiziert bei Kindern, Jugendlichen und Erwachsenen, bei denen mit hoher Wahrscheinlichkeit täglich und zu unvorhersehbaren Zeiten lebensbedrohliche gesundheitliche Situationen auftreten können. Die AKI-Maßnahmen erlauben nach dem sozialgesetzlich formulierten Grundsatz „ambulant vor stationär“ den überwiegenden Verbleib der Versicherten im häuslichen Umfeld, also im familiären Bereich bzw. in der vertrauten Umgebung. Darüber hinaus soll durch die AKI eine möglichst komplette Vermeidung jeglicher stationärer Krankenhausaufenthalte erreicht werden. Bereits im Rahmen des zum Ende der akuten Krankenhausbehandlung implementierten abschließenden Entlassmanagements kann AKI zur Unterstützung einer sektorenübergreifenden Versorgung über einen Zeitraum von bis zu sieben Kalendertagen aus der stationären Versorgung heraus verordnet werden und gilt als Erstverordnung. Für Versicherte die nicht unmittelbar vor der AKI-Versorgung stationär behandelt worden sind, kann durch die verordnende Vertragsärztin oder den verordnenden Vertragsarzt eine Erstverordnung von bis zu fünf Wochen ausgestellt werden. Grundsätzlich hat die Vertragsärztin/der Vertragsarzt sich bei der Folgeverordnung über den Erfolg der verordneten Maßnahme zu vergewissern. Die Folgeverordnung kann anschließend längstens für sechs Monate ausgestellt werden.

Voraussetzungen sind jeweils die mustergültige Erfüllung der vertragsärztlichen, für die Leistungsentscheidung obligaten Formalia sowie die Sicherstellung einer umfassenden Behandlungspflege, die zur Erreichung des vereinbarten Ziels der vertragsärztlichen Behandlung erforderlich ist. Hier steht die Zusammenarbeit aller an der medizinischen und pflegerischen Versorgung beteiligten ärztlichen und nichtärztlichen Leistungserbringer im Vordergrund, koordiniert durch besonders qualifizierte verordnende Vertragsärztinnen und Vertragsärzte. Eine sachgerechte Durchführung der AKI durch entsprechend qualifiziertes Pflegefachpersonal, insbesondere zur Sicherstellung der medizinischen und pflegerischen Versorgung am Leistungsort, erscheint als eine Grundvoraussetzung unabdingbar. Ziel der vorliegenden Arbeit ist es, hierfür die in Hessen geübte richtlinienkonforme Praxis vorzustellen und über erste Auswertungen sowie darauf basierend handlungsleitende Erfahrungen zu berichten.

## Wesentliche Regelungen

In Verbindung mit der – den § 37c SGB V operationalisierenden – Richtlinie des Gemeinsamen Bundesausschusses (G-BA) über die Verordnung von außerklinischer Intensivpflege (Außerklinische Intensivpflege-Richtlinie/AKI-RL) wurden zudem Rahmenempfehlungen nach § 132 l Abs. 1 SGB V zur Versorgung mit außerklinischer Intensivpflege zwischen dem GKV-Spitzenverband und den maßgeblichen Vertretungen der Leistungserbringer auf Bundesebene vereinbart. Diese traten mit einer einjährigen Übergangszeit zum 01.07.2023 in Kraft.

Anspruch auf AKI nach § 37c SGB V haben Versicherte mit einem besonders hohen Bedarf an medizinischer Behandlungspflege. Dieser liegt vor, wenn die ständige Anwesenheit einer geeigneten Pflegefachperson zur individuellen Kontrolle und Einsatzbereitschaft oder ein vergleichbar intensiver Einsatz einer solchen erforderlich ist. Die AKI als wichtiger Bestandteil der Gesundheitsversorgung konzentriert sich demnach auf die Betreuung von Versicherten mit komplexen Pflegebedürfnissen, die zwar nicht im Krankenhaus behandelt werden müssen, aber auch nicht mehr in der Lage sind, sich selbständig zu versorgen. Die vertragsärztlich zu verordnende AKI kann an folgenden Leistungsorten erbracht werden, wobei berechtigten Wünschen der Versicherten zu entsprechen ist:

- in der eigenen Häuslichkeit mit Versorgung durch ambulante Intensivpflegedienste in meistenteils individueller 1:1-Betreuung,
- in stationären Pflegeeinrichtungen mit intensivpflegerischer Versorgung,
- in qualitätsgesicherten Intensivpflege-Wohneinheiten,
- in Einrichtungen der Hilfe für behinderte Menschen
- sowie an weiteren geeigneten Orten, wie z. B. Schulen, Kindergärten und Werkstätten.

In der am 15.09.2023 in Kraft getretenen AKI-RL sind Leistungsinhalte, Voraussetzungen und Maßgaben der Verordnung, Vorgaben der Potenzialerhebung zur Reduzierung der Beatmungszeit bis hin zur vollständigen Beatmungsentwöhnung (Weaning) oder Entfernung einer Trachealkanüle (Dekanülierung) sowie die Qualifikation der potenzialerhebenden und auch verordnenden Ärztinnen und Ärzte dezidiert geregelt 3 . Ein Anspruch auf AKI besteht nur dann, wenn die oder der Versicherte die außerklinische Intensivpflege nicht selbst durchführen kann. Für die AKI sind ab dem 31.10.2023 ausschließlich mittels der folgenden drei Musterformulare auszustellen:

- 62 A (Potenzialerhebung verpflichtend mit neuer Regelung ab dem 01.07.2025),
- 62B (eigentliche Verordnung),
- 62 C (Behandlungsplan).

Für die alleinige **vertragsärztliche Verordnung** von AKI (Muster 62B und 62 C) können sich Hausärztinnen und Hausärzte – nach dem (niedrigschwelligen) Nachweis von „Kompetenzen im Umgang mit beatmeten oder trachealkanülierten Versicherten“ – gegenüber der Kassenärztlichen Vereinigung (KV) als Verordner/-innen qualifizieren. „Die Genehmigung ist auf Antrag zu erteilen, wenn die Antragstellerin oder der Antragsteller nachweist, dass sie oder er die vorgenannten Voraussetzungen erfüllt oder die Absicht erklärt, sich diese innerhalb von sechs Monaten anzueignen und nachzuweisen…“ Die Verordnung von AKI kann aber auch durch „besonders qualifizierte“ Ärztinnen und Ärzte (s. u.) erfolgen, diese sind per se als Verordnende qualifiziert.

Für eine **Genehmigung zur Potenzialerhebung** nach Muster 62 A ist die Zusatzbezeichnung Intensivmedizin oder Pneumologie oder – zusätzlich zur fachärztlichen Qualifikation (wie z. B. Innere Medizin, Anästhesiologie, Chirurgie, Neurochirurgie, Neurologie, Kinder- und Jugendmedizin sowie weitere Fachärztinnen und Fachärzte) – eine besondere Qualifikation nachzuweisen (die je nach Spezialisierung eine zwischen 6- und 18-monatige „einschlägige Tätigkeit in der prolongierten Beatmungsentwöhnung“ bedingt; verbindliche Details dazu siehe AKI-RL §§ 8 und 9).

Die **Rahmenempfehlungen** nach § 132 l Abs. 1 SGB V regeln u. a. die aufgabenspezifische Qualifikation der verantwortlichen Pflegefachpersonen zur Versorgung von AKI-Versicherten, personelle und strukturelle Anforderungen an Wohneinheiten sowie an vollstationäre Pflegeeinrichtungen, Einzelheiten zu Inhalt und Umfang der Zusammenarbeit von Leistungserbringen mit Verordnern, Krankenhäusern und nichtärztlichen Leistungserbringern. Ebenfalls geregelt wurden Maßnahmen und Anforderungen bzgl. der Qualitätssicherung, des einrichtungsinternen Qualitätsmanagements, der Fortbildung sowie der Pflegeprozesse und -dokumentation 4 . Letztendlich ist festzuhalten, dass nur qualitätsgeprüfte Pflegedienste AKI-Leistungen durchführen dürfen.

## Genehmigung durch die Krankenkasse

Die Feststellung, ob die Voraussetzungen einer AKI im Einzelfall erfüllt sind, wird durch die Krankenkasse (KK) getroffen – nach (in der Regel verpflichtend vorangegangener) persönlicher Begutachtung des Versicherten am Leistungsort durch den Medizinischen Dienst (MD) 5 . Diese Begutachtung dient auch der Klärung, ob die medizinische und pflegerische Versorgung sichergestellt ist und ggf. ein Weaning- bzw. Dekanülierungspotenzial hinsichtlich Beatmung bzw. vorhandener Trachealkanüle vorliegt. Die Feststellung der KK ist von dieser spätestens jährlich auf der Grundlage einer erneuten persönlichen Begutachtung durch den MD zu überprüfen. Werden Leistungen der AKI nicht oder nicht in vollem Umfang genehmigt, hat die Krankenkasse dem verordnenden Vertragsarzt sowie dem Versicherten die Begründung mitzuteilen.

## Begutachtung durch den MD

Allgemeine Grundlage der Begutachtung durch den MD ist das konzeptionelle und begriffliche Bezugssystem – die Rahmentheorie des biopsychosozialen Modells mit der daraus abgeleiteten International Classification of Functioning, Disability and Health (Internationale Klassifikation der Funktionsfähigkeit, Behinderung und Gesundheit, ICF) – der Weltgesundheitsorganisation (WHO). Spezifische Grundlage der bundesweit verpflichtenden, aber föderal organisierten sozialmedizinischen MD-Begutachtungen bildet die „ **Begutachtungsanleitung** Richtlinie des Medizinischen Dienstes Bund … Außerklinische Intensivpflege nach § 37c SGB V ( **BGA AKI** )“. Diese wurde von allen einschlägigen Organisationen und Verbänden konsentiert und vom Bundesministerium für Gesundheit (BMG) abschließend genehmigt 6 . Die komplexe sozialmedizinische Begutachtung bedarf dabei der Heranziehung, Bewertung und Beurteilung begutachungsrelevanter Unterlagen und Befunde mit dem Hauptaugenmerk auf der sozialmedizinischen Epikrise (Box) 7 . Grundsätzlich erfolgt die persönliche Inaugenscheinnahme vor Ort in Hessen aktuell immer durch besonders qualifizierte und erfahrene ärztliche Gutachter und Gutachterinnen, die alle über fachärztliche Kompetenzen im Umgang mit diesen Versicherten und zusätzlich über sozialmedizinische Expertise verfügen. Zudem kann es ggf. sinnvoll sein, punktuell weitere Fachärztinnen und Fachärzte mit speziellen Qualifikationen bzw. auch spezielle Fachkräfte (z. B. Atmungstherapeutinnen und -therapeuten) konsiliarisch zu beteiligen. Die sozialmedizinische Beurteilung wird in jedem Einzelfall von einem ärztlichen Gutachter oder einer ärztlichen Gutachterin erstellt, auch wenn zusätzlich eine Pflegefachperson eingesetzt wird. Beim MD Hessen wird die Begutachtung am Leistungsort grundsätzlich im kombinierten Team aus einer Fachärztin/einem Facharzt und einer Pflegefachperson durchgeführt („Tandemprinzip“).

**BOX**

Begutachtungsrelevante Unterlagen bzw. Informationen in aktueller Version:

- vertragsärztliche Verordnung (Muster 62B, 62 C)
- fachärztlicher Befund zur Potenzialerhebung (Muster 62 A)
- Entlassmanagement: Überleitungsbogen/(vorläufiger) Klinikbericht
- Arzt-/Befundberichte zur aktuellen Erkrankung inkl. ggf. zur Polysomnographie
- Berichte zur Physio-, Logo- bzw. Ergotherapie
- Berichte der Spezialambulanzen und Sozialpädiatrischen Zentren
- pflegerische Dokumentationen/Protokolle der letzten 4 Wochen anlässlich der Vitalfunktionsbeobachtung, Beatmungs-, Absaug- und Krampfereignisse
- Bedarfsmedikation (Medikamentenplan)
- Leistungsauszüge der Krankenkasse

Die Abfassung des Gutachtens hat nach der von der Begutachtungsanleitung für alle Gutachterinnen und Gutachter verbindlich vorgegeben Gliederung inkl. einer Erläuterung des Ergebnisses zu erfolgen. Im Fokus steht, ob

- die sozialmedizinischen Voraussetzungen für die Leistung der AKI erfüllt sind und
- die medizinische und pflegerische Versorgung am Leistungsort sichergestellt ist (oder diese durch entsprechende Nachbesserungsmaßnahmen in angemessener Zeit sichergestellt werden kann). Unterschieden werden muss dabei zwischen Behandlungspflege nach § 37 sowie der neu hinzugefügten AKI nach § 37c als Leistung der Krankenkasse nach dem SGB V und im Rahmen der Begutachtungssituation. Die qualitätsgesicherten Gutachten enthalten die Beurteilung wesentlicher Aspekte zu Therapieoptimierung, Beatmungsentwöhnung oder Dekanülierungspotenzial. Neben der erläuterten Bewertung der AKI-Voraussetzungen als „erfüllt“, „nicht erfüllt“ bzw. „eingeschränkt oder teilweise erfüllt“ finden sich im MD-Gutachten auch sozialmedizinische Empfehlungen.

## Erhebungsmethode

Die AKI-Gutachten des MD Hessen werden nach bundeseinheitlich verbindlichen Kriterien standardisiert qualitätsgesichert 8 . Wesentliche Parameter der einzelnen im Rahmen einer persönlichen Inaugenscheinnahme vor Ort erstellten AKI-Gutachten werden fortlaufend statistisch anonymisiert in einer Datenbank erfasst. Darauf basierend erfolgte eine retrospektive Sekundärdatenanalyse der durch ein spezialisiertes Tandemteam aus Facharzt/Fachärztin plus Fachpflegekraft erstatteten Gutachten des MD Hessen anlässlich der vertragsärztlich verordneten AKI im Zeitraum von Oktober 2023 bis März 2025. Verbindliche Grundlage bildete die vom Bundesministerium für Gesundheit genehmigte Begutachtungsanleitung des MD Bund.

## Ergebnisse häuslicher Begutachtungen durch den MD Hessen

In den ersten 17 Monaten seit dem verbindlichen Beginn der persönlichen Inaugenscheinnahmen am 31.10.2023 wurden inkl. einiger Wiederholungsbegutachtungen 1615 Vor-Ort-Begutachtungen bei insgesamt 1340 Versicherten durchgeführt (274 Versicherte wurden in diesem Zeitraum zum zweiten Mal besucht, ein Versicherter bereits dreimal). Der Alters-Median aller AKI-Versorgten lag bei 56 Jahren (8 Jahre bei Kindern bzw. Jugendlichen; 63 Jahre bei Erwachsenen). Bei den Erwachsenen waren 60,1% männlichen und 39,9% weiblichen Geschlechts, bei Kindern war der geschlechtsspezifische Unterschied weniger ausgeprägt, nämlich 52,1% vs. 47,9% ( Tab. 1 ). Der größte Teil der Versicherten (56,9%) wurde in der eigenen Häuslichkeit versorgt, gefolgt von vollstationärer Pflege (23,0%) und der Versorgung in einer Intensivpflege-Wohneinheit (14,6%) bzw. Kindergärten, Schulen, Werkstätten, etc. (5,4%) ( Tab. 2 ).

**Table:** **Tab. 1** Sozialmedizinische Prüfung der Verordnung von außerklinischer Intensivpflege durch den Medizinischen Dienst Hessen im Zeitraum 10/2023–04/2025: Altersverteilung der begutachteten Fälle.

Altersverteilung		
Alter (Mittelwert/Median)	Anzahl (Anteil)	altersgruppen-spezifischer Anteil
**0–18 Jahre (8/8)**	**399 (24,7%)**	**100,0%**
männlich	208 (12,9%)	52,1%
weiblich	191 (11,8%)	47,9%
divers	0 (0%)	0%
**˃ 18 Jahre (60/63)**	**1216 (75,3%)**	**100,0%**
männlich	731 (45,3%)	60,1%
weiblich	485 (30,0%)	39,9%
divers	0	0%
**0–97 Jahre (47/56)**	**1615 (100%)**	**100,0%**
männlich	939 (58,1%)	58,1%
weiblich	676 (41,9%)	41,9%
divers	0 (0%)	0%

**Table:** **Tab. 2** Sozialmedizinische Prüfung der Verordnung von außerklinischer Intensivpflege durch den Medizinischen Dienst Hessen im Zeitraum 10/2023–04/2025: Versorgungsorte der begutachteten Fälle.

Versorgungsort					
Alter	Häuslichkeit	Pflegeheim	Wohngruppe	KiTa/Schule/Werkstatt	Gesamt
**0–18 Jahre**	243 (15,0%)	69 (4,3%)	9 (0,6%)	78 (4,8%)	399 (24,7%)
**˃ 18 Jahre**	677 (41,9%)	303 (18,8%)	226 (14,0%)	10 (0,6%)	1216 (75,3%)
**0–97 Jahre**	920 (56,9%)	372 (23,0%)	235 (14,6%)	88 (5,4%)	1615 (100,0%)

Hervorzuheben ist der hohe Anteil der Begutachtungen bei tracheotomierten erwachsenen Patientinnen und Patienten von 79,2% der Gesamtpopulation im Unterschied dazu bei dem der Subpopulation „Alter unter 18 Jahren“ mit 10,1%. 448 tracheotomierte Versicherte (34,1%) wurden zusätzlich durchgängig oder intermittierend invasiv beatmet ( Tab. 3 ).

**Table:** **Tab. 3** Sozialmedizinische Prüfung der Verordnung von außerklinischer Intensivpflege durch den Medizinischen Dienst Hessen im Zeitraum 10/2023–04/2025: Art der respiratorischen Versorgung der begutachteten Fälle (TS=Tracheostoma).

Art der respiratorischen Versorgung				
Alter	TS mit Beatmung	TS ohne Beatmung	Beatmung ohne TS	Gesamt
**0–18 Jahre**	79 (6,0%)	54 (4,1%)	69 (5,3%)	202 (15,4%)
**˃ 18 Jahre**	369 (28,1%)	671 (51,1%)	72 (5,4%)	1112 (84,6%)
**0–97 Jahre**	448 (34,1%)	725 (55,2%)	141 (10,7%)	1314 (100,0%)

Bis zum 31.03.2025 war bereits bei 703 (53,5%) der Gutachtenaufträge aller invasiv und nicht-invasiv beatmeten sowie tracheotomierten Patientinnen und Patienten die gesetzlich vorgesehene Potenzialerhebung durchgeführt worden, 21,8% hiervon mit dem Ergebnis eines aktuell oder perspektivisch positiven Potenzials ( Tab. 4 5 ). In 611 Begutachtungsfällen (46,5%) war diese Potentialerhebung damals noch ausstehend. In insgesamt 301 Fällen (18,7%) war aufgrund des Erkrankungsbildes (keine Beatmung, keine Trachealkanüle) eine Potenzialerhebung nicht erforderlich.

**Table:** **Tab. 4** Sozialmedizinische Prüfung der Verordnung von außerklinischer Intensivpflege durch den Medizinischen Dienst Hessen im Zeitraum 10/2023–04/2025: Vorliegen des Musterformulars 62 A zur Weaning-Potenzialerhebung.

Musterformular 62 A zur Weaning-Potenzialerhebung			
Alter	vorliegend	nicht vorliegend	Gesamt
**0–18 Jahre**	90 (6,8%)	112 (8,5%)	202 (15,4%)
**>18 Jahre**	613 (46,7%)	499 (37,9%)	1112 (84,6%)
**0–97 Jahre**	703 (53,5%)	611 (46,5%)	1314 (100,0%)

**Table:** **Tab. 5** Sozialmedizinische Prüfung der Verordnung von außerklinischer Intensivpflege durch den Medizinischen Dienst Hessen im Zeitraum 10/2023–04/2025: Ergebnis der vertragsärztlichen Potenzialerhebung nach Musterformular 62 A.

Ergebnis der vorliegenden Potenzialerhebung nach Musterformular 62 A			
Alter	aktuell/perspektivisch vorhanden	nicht vorhanden	Gesamt
**0–18 Jahre**	19 (2,7%)	71 (10,1%)	90 (12,8%)
**>18 Jahre**	134 (19,1%)	479 (68,1%)	613 (87,2%)
**0–97 Jahre**	153 (21,8%)	550 (78,2%)	703 (100,0%)

In rund 8,3% aller Begutachtungsfälle konnte die Notwendigkeit einer AKI sozialmedizinisch nicht nachvollzogen werden, bei 88,4% war diese aber ganz oder teilweise nachvollziehbar ( Tab. 6 ). Bei stichprobenartig analysierten 90 Gutachten war gutachterlich festzustellen, dass das Ergebnis der dokumentierten Potenzialerhebung gutachterlicherseits in nur 44% als plausibel nachvollzogen werden konnte.

**Table:** **Tab. 6** Sozialmedizinische Prüfung der Verordnung von außerklinischer Intensivpflege durch den Medizinischen Dienst Hessen im Zeitraum 10/2023–04/2025: Ergebnisse. der gutachterlichen Beurteilung der sozialmedizinischen Voraussetzungen für eine Leistungsgewährung seitens der Krankenkasse.

Begutachtungsergebnisse				
Alter	sozialmedizinische Voraussetzungen für Leistungsgewährung			Sonstiges
	erfüllt	eingeschränkt oder teilweise erfüllt	nicht erfüllt	
**0–18 Jahre**	266 (16,5%)	78 (4,8%)	51 (3,2%)	4 (0,2%)
**˃ 18 Jahre**	857 (53,1%)	227 (14,1%)	82 (5,1%)	51 (3,2%)
**0–97 Jahre**	1123 (69,5%)	304 (18,8%)	133 (8,3%)	55 (3,4%)

## Limitationen

Die Erhebung erfolgte von Anfang an gleich mit der Frühphase der umfassend neu geregelten AKI-Begutachtung, also unmittelbar nach Inkrafttreten der Begutachtungsanleitung als Richtlinie des MD Bund. Hier könnten sich, z. B. aufgrund der Auswirkungen möglicher adaptiver gesetzlicher Vorgaben, des Endes der Übergangsregelung, der Adaption in der Verordnungspraxis und Potentialerhebungsroutine sowie zweier angemeldeter AKI-Leitlinienprojekte, zum einen bezogen auf Versicherte mit respiratorischen Erkrankungen sowie andererseits diejenigen mit Erkrankungen des Nervensystems oder der Muskulatur, modifizierte Erkenntnisse ergeben. Die zwar hier nur für ein einziges Bundesland momentan vorliegenden umfänglichen Daten aus sämtlichen im genannten Zeitraum erstatteten AKI-Gutachten können allerdings, da Hessen ein Flächenland mit zwei wirtschaftsstarken Metropolregionen und bundesweit mehr als 7 Prozent aller GKV-Versicherten darstellt, bezogen auf die gegenwärtige epidemiologische Situation in Deutschland als durchaus repräsentativ betrachtet werden.

## Fazit

Seit dem 31.10.2023 hat die sozialmedizinische Begutachtung anlässlich einer Verordnung von AKI gesetzlich einmal jährlich verpflichtend am Leistungsort auf der Grundlage einer persönlichen Inaugenscheinnahme durch den MD zu erfolgen. Dies ermöglicht ab diesem Zeitpunkt allen Beteiligten ein viel individuelleres und differenzierteres Bild der einzelnen Versicherten und ihrer speziellen Situation. Im Vordergrund steht bei einer Vor-Ort-Begutachtung neben der Feststellung der grundsätzlichen sozialmedizinischen Notwendigkeit einer AKI-Versorgung immer auch die Sicherstellung der adäquaten medizinischen und pflegerischen Versorgung, so wie auch hinsichtlich eines bislang nicht hinreichend ausgeschöpften Weaning- oder Dekanülierungs-Potenzials 9 10 . Beim MD Hessen wird diese besonders profunde und umfängliche Art der Begutachtung vor Ort grundsätzlich im Zweierteam aus Fachärztin bzw. Facharzt und einer Pflegefachperson durchgeführt. Diese Gutachterinnen und Gutachter stehen hierbei sowohl den Versicherten und ihren Angehörigen als auch den beauftragenden Krankenkassen als unabhängige Beraterinnen und Berater für die Überprüfung einer sozialgesetz- und richtlinienkonformen qualitativen Versorgung zur Verfügung.

Die persönliche Begutachtung vor Ort stellt kontextabhängig für die Versicherten und ihre Angehörigen eine oft ungewohnte und von Stress begleitete Situation dar. Hier können eine umsichtige und wertschätzende Inaugenscheinnahme des privaten Umfelds und die sorgsame Evaluation des persönlichen Versorgungsnetzwerks eine eigentlich als indiskret empfundene Situation entschärfen. So kann das bereits bei der Pflegeeinzelfallbegutachtung beschriebene Phänomen der Verwundbarkeit von Personen in Face-to-Face-Interaktionen (zurückzuführen auf die Explizitheit und Taktlosigkeit eines epistemisch konturierten Verfahrens) vermieden werden. Trotzdem wird infolge des sozial exponierten und konkretisierten Bedürftigkeitserlebnisses die sozialmedizinische Begutachtung oft zu einer „persönlichen und moralischen Angelegenheit“ 11 . Dementsprechend wird diese Begutachtungssituation auch seitens der Gutachterinnen und Gutachter nicht selten als deutlich emotionale Belastung empfunden.

Die entscheidungsrelevante Basis jeder AKI-Begutachtung ist immer das mustergültige Vorliegen einer aussagekräftigen Verordnung sowie – nach dem Ende der Übergangsregelung definiert – einer regelgerecht dokumentierten Potenzialerhebung 12 . Im Bundesland Hessen ist die richtlinienkonforme Umsetzung flächendeckend bereits jetzt zu großen Teilen realisierbar. So gab es zum 09.04.2025 von der KV Hessen zur AKI-Verordnung 197 und zur Potenzialerhebung insgesamt 35 autorisierte Vertragsärztinnen und -ärzte. Die erforderliche (niedrigschwellige) Qualifikation bei den von der KV Hessen zu autorisierenden Verordnerinnen und Verordnern sollte mittelfristig eine Ausweitung dieses Kreises bewirken. Ob bei den Potenzialerheberinnen und -erhebern ein ähnlicher Effekt zum Tragen kommt bleibt abzuwarten.

Festzuhalten bleibt, dass die konsequente Ausschöpfung der vorhandenen Möglichkeiten zur Beatmungsentwöhnung und Dekanülierung einer engen Kooperation aller Beteiligten (entlassende Kliniken, Verordner, Potenzialerheber, Krankenkassen, Pflegedienste, Atemtherapeuten, Heilmittelerbringer, Angehörige bzw. Betreuer) bedarf. Essenziell dabei bleibt, im Rahmen der sozialgesetzlich verpflichtenden Verantwortung für die Gesamtkoordination seitens der verordnenden Ärzte/Ärztinnen eine zuverlässige intensivpflegerische Versorgung dieser vulnerablen Gruppe zu gewährleisten. Der Stellenwert der verpflichtend und lege artis durchzuführenden Potenzialerhebung bedarf seit dem Ende der Übergangsregelung mit der Maßgabe der diesbezüglichen vollständigen Erfassung tracheotomierter und/oder beatmeter Versicherter zukünftig weiterer Analysen.
